# Exploring gait velocity as a predictor of cardiometabolic disease risk in young adults

**DOI:** 10.3389/fspor.2024.1365717

**Published:** 2024-03-07

**Authors:** Tanner Thorsen, Nuno Oliveira, Austin Graybeal, Jon Stavres

**Affiliations:** School of Kinesiology and Nutrition, The University of Southern Mississippi, Hattiesburg, MS, United States

**Keywords:** walking, walking speed, gait analysis, metabolic syndrome, young adults

## Abstract

**Background:**

The association between poor gait and functional movement ability and metabolic syndrome (MetS) has been well established in older adults. A continuous cardiometabolic risk score, MetS_index_, may more easily identify individuals at risk for cardiometabolic disease who do not yet meet the stringent criteria for a formal MetS diagnosis. Although the association between MetS and gait velocity is well established in older adults, no such relationship has been identified in younger adults; a group experiencing a rapid increase in the development of MetS.

**Methods:**

MetS_index_ was determined for 21 young adults using standard procedures. Gait velocity was measured as participants completed a ten-meter walk test. Spatiotemporal parameters of gait were also derived using a motion capture system. Simple linear regression was used to determine the relationship between MetS_index_ and gait velocity, as well as MetS_index_ and spatiotemporal parameters of gait.

**Results:**

There was a large inverse relationship between MetS_index_ and gait velocity. A large inverse relationship was also observed between MetS_index_ and cadence, and a large positive relationship was observed between stance time and double limb support time.

**Conclusions:**

Gait velocity slows in young adults who do not necessarily meet the criterion for positive diagnosis of MetS—but demonstrate an increased risk for MetS and cardiovascular disease through higher MetS_index_ scores. The mechanism underlying reduced gait velocity may be fewer, but not shorter steps. Determining easy-to-use surrogates of MetS (e.g., gait velocity) may help combat the growing prevalence of MetS by increasing access to preventative approaches.

## 1 Introduction

The association between metabolic syndrome (MetS) and poor gait and functional movement ability has been well established in older adults (1–4). Gait velocity is a well-known indicator of functional decline and is often used as a simple and reliable measure of the functional capacity of older adults (5). Some have even suggested that gait velocity be considered “the functional” or “sixth vital sign” and research suggests that it is highly sensitive to change (with advanced age, training status, disease, cognition, etc.) similar to heart rate or blood pressure (6). Reduced gait velocity, for example, has been shown to be an important predictor of metabolic complications, with 0.1 m/s reductions in gait velocity associated with an 8% increase in cardiovascular disease (3, 4, 7). To that end, an increasing body of research has used gait analysis to predict metabolic complications including MetS, type II diabetes, and cardiovascular disease (CVD) (2, 7). A recent meta-analysis and systematic review has even recommended the inclusion of gait velocity as a predictor of CVD and mortality (7).

Currently, MetS is diagnosed based on the presence of at least three of the following cardiometabolic risk factors: (1) excess abdominal adiposity, (2) elevated blood pressure, (3) hyperglycemia, (4) low HDL cholesterol, and (5) elevated serum triglycerides (8). While the current MetS diagnosis employs a dichotomous classification system (i.e., “yes” or “no”), research from our group and others suggests this diagnostic approach does not adequately identify all individuals with an elevated risk of cardiometabolic disease, particularly in young adults (9). For instance, members of our research team recently collected data from 104 young adults, 55 (64.7%) of whom presented with at least one cardiometabolic risk factor and 19 (18.3%) met the criteria for MetS (10).

Recently, a continuous cardiometabolic risk score (MetS_index_) has been developed and may be equipped to identify individuals at risk for cardiometabolic disease who do not yet meet the stringent criteria for a formal MetS diagnosis (9). This score accounts for the severity of each of the five MetS risk factors, while also adjusting for race and biological sex. The final outcome of this score is a single value (interpreted as a z-score) that compares an individual's cardiometabolic disease risk to that of the general US adult population, with a more negative score indicating less cardiometabolic disease risk and a positive score indicating increased cardiometabolic disease risk. This continuous MetS_index_ allows researchers and clinicians to evaluate cardiometabolic disease risk along a continuum, offering the potential to preemptively identify disease risk in individuals not yet meeting the defined criteria for MetS. Therefore, MetS_index_ is more relevant for populations that might be at risk of developing cardiometabolic disease, such as young adults.

Although the association between MetS and gait velocity is well established in older adults, no such relationship has been identified between gait velocity and MetS_index_ in younger adults. Understanding this association may be highly valuable in the early detection and prevention of MetS, allowing more time for targeted interventions. Thus, we sought to explore the relationship between gait velocity and MetS_index_ in younger adults. We hypothesized that a negative relationship would exist between gait velocity and MetS_index_ scores in younger adults. As such, our primary variables of interest were MetS_index_ and gait velocity. We also analyzed the spatiotemporal parameters of gait as explanatory variables defining the underlying mechanistic changes in gait velocity we hypothesized.

## 2 Method

Twenty-one participants were prospectively recruited to attend 2 laboratory sessions. Inclusion criteria included adults between the ages of 18–29, and free from any lower extremity injury, surgery, or disorder that would affect gait (Male: 4; Female: 17; Height:165.5 ± 8.7 cm; Weight: 70.6 ± 18.2 kg; BMI: 22.1 ± 2.0 kg/m^2^), and all participants provided written informed consent approved by the local Institutional Review Board.

During the first visit, MetS_index_ was determined using previously established methodology (11, 12). Height was measured using a stadiometer and weight was collected using a calibrated digital scale (SECA, Hamburg, Germany).Waist circumference was collected by a trained investigator at the level of the iliac crest using a flexible aluminum tape measure per the guidelines for evaluating abdominal obesity put forth by the National Cholesterol Education Program (NCEP) Adult Treatment Panel III (ATP-III). Resting blood pressure was measured via an automated sphygmomanometer. Approximately 40 µl of capillary blood (serum) were collected into lithium heparin-lined capillary pipettes to measure fasting blood glucose (%CV: 4.5–6.2), HDL cholesterol (%CV: 3.3–4.9), and triglycerides (%CV: 1.6–3.6) using a capillary blood analyzer (Cholestech LDX; Abbott Laboratories, Chicago, IL). These variables served as the requisite inputs for the MetS_index_ algorithm (9). MetS_index_ was calculated from existing sex- and race-specific equations (9) using the five MetS risk described above with the exclusion of diastolic blood pressure. Specifically, MetS_index_ generates a continuous risk score that represents an estimate of MetS severity as opposed to the traditional binary approach that employs rigid cutoffs for MetS classification. The final estimate produced by the equation is reflective of a z-score, where negative scores indicate decreased MetS severity, and positive scores indicate increased MetS severity. Importantly, these equations have been cross validated in separate samples and have shown to be related to related cardiometabolic abnormalities (9).

During the second visit, participants completed three trials of the ten-meter walk test (10MWT) along a level walkway. Gait velocity was measured during the middle 6 meters of the walkway using 2 photocell timing gates (Blue, Dashr, Lincoln, NE, USA). The mean velocity from all three trials was computed and used for analysis. Spatiotemporal parameters of gait were determined using a standard bilateral lower extremity marker set to record lower extremity kinematics during each 10MWT with a 10-camera motion capture system (240 Hz, Qualisys, Göteborg, Sweden). Spatiotemporal parameters were determined during the stance phase of each step, defined between heel-strike and toe-off of the right leg, specifically when the ground reaction force measured by the force plate exceeded a threshold of 10 Newtons (heel-strike) or fell below a threshold of 10 Newtons (toe-off) (1,200 Hz, American Mechanical Technology Inc. Watertown, MA, USA). All Spatiotemporal parameters of gait were calculated using Visual 3D (version 6.0, C-Motion, Germantown, MD, USA).

The relationship between MetS_index_ and variables of interest was assessed using simple linear regression techniques. An alpha level of.05 was established *a priori* and the strength of regression was determined using cutoffs of 0.2 = small, 0.5 = moderate, and 0.8 = strong (13). All statistical analyses were performed using SPSS (version 28, IBM, Chicago, IL).

## 3 Results

**Figure 1:**
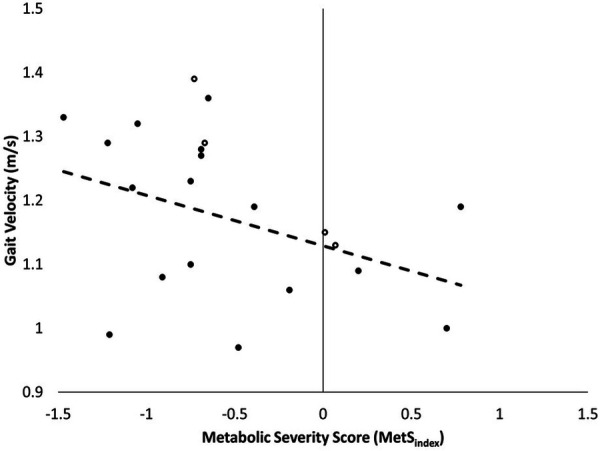
Correlation between metS_index_ and gait velocity in younger adults (*n* = 21), with females represented with solid markers and males with outlined markers.

Participant demographic, anthropometric, and metabolic information are presented in Table 1. There was a large, inverse relationship between MetS_index_ and gait velocity in younger adults (Figure 1, Table 2). Furthermore, a large association was observed between MetS_index_ and cadence, indicating participants walked with reduced cadence (steps per minute) as MetS_index_ increased. Stance time and double limb support time demonstrated large positive associations with MetS_index_, indicating participants increased contact time during stance as MetS_index_ increased (Table 2).

**Table 1:** Participant characteristics for the sample of *N* = 21 young adults.

Sex	*N* (%)
Male	4 (19.0%)
Female	17 (81.0%)
Race (*n*)	
White	9 (42.9%)
Black	12 (57.1%)
Ethnicity	
Hispanic	1 (4.8%)
	Mean ± SD
Age (year)	22.1 ± 2.0
Height (cm)	165.5 ± 8.7
Weight (kg)	70.6 ± 18.2
BMI (kg/m^2^)	25.5 ± 4.9
Waist circumference (cm)	84.2 ± 14.5
Systolic blood pressure (mmHg)	115.2 ± 12.5
Diastolic blood pressure (mmHg)	78.3 ± 8.3
Triglycerides (mg/dl)	102.1 ± 75.7
HDL cholesterol (mg/dl)	53.1 ± 13.2
Fasting blood glucose (mg/dl)	89.6 ± 7.2
MetSindex	−0.57 ± 0.59
	*N* (%)
Metabolic syndrome	2 (9.5%)
Abdominal obesity	6 (28.6%)
Hypertension	7 (33.3%)
Hypertriglyceridemia	3 (14.3%)
Dyslipidemia	9 (42.9%)
Hyperglycemia	3 (14.3%)

Data are presented as mean ± standard deviation or as *n* (% of the column total).

**Table 2:** Spatiotemporal parameters of gait, beta coefficients (*β*), correlation coefficients (*r)*, and *P*-values (*P*) between each spatiotemporal parameter of gait and metS_index_.

Variable	Mean ± s.d.	*β*	*r*	*P*
Gait velocity (m/s)	1.16 ± 0.15	−2.942	−0.524	.**026**
Cadence (SPM)	112.47 ± 9.18	−0.037	−0.576	.**012**
Step length (m)	0.65 ± 0.04	−6.659	−0.418	.084
Step width (m)	0.21 ± 0.04	1.740	0.104	.681
Stance time (s)	0.66 ± 0.06	6.069	0.609	.**007**
Double support time (s)	0.24 ± 0.05	8.736	0.666	.**003**

Bold indicates statistical significance.

## 4 Discussion

These results indicate a strong, inverse relationship exists between young adults who do not necessarily meet the criterion for positive MetS diagnosis—but demonstrate an increased risk for MetS and CVD through a positive MetS_index_ score—and self-selected gait velocity (Figure 1). Because MetS is likely under classified in many young adults using the current diagnostic standards, using the MetS_index_ may aid in the early detection and prevention of modifiable risk factors in young adults, ultimately reducing their long-term risk of developing cardiometabolic diseases and improving their overall longevity. Despite the higher incidence of positive MetS risk factors, primarily driven by a prevalence of obesity, adults are less likely to receive the routine health evaluations necessary for the early detection of MetS (14, 15). Given that obesity is highly associated with the early development of MetS (which may be more effectively determined using MetS_index_), discovering easy-to-use surrogates that can leverage this relationship (e.g., gait velocity) may help combat the growing prevalence of MetS by increasing access to preventative approaches.

In this current study, dyslipidaemia, hypertension, and abdominal obesity were the most prevalent metabolic variables—all of which have been associated with reduced gait velocity in older adult populations. Ashan et al. (2023) reported reduced gait velocity in hypertensive older adults compared to normotensive older adults (16), and a recent longitudinal study reported a greater rate of yearly decline in gait velocity with hypertensive patients compared to normotensive patients (17). In addition, the negative effects of obesity have been clearly demonstrated in adults. Hulens et al., for example, reported a significant reduction of 18% in gait velocity during a 6-minute walk test in obese and morbidly obese adult women (18). The results of this current study support the existing body of literature and demonstrate the negative impact of common metabolic dysfunction variables on gait velocity in older age are also manifest in younger adults. As such, monitoring gait velocity beginning earlier in adulthood may serve as an accessible and inexpensive indicator of underlying metabolic dysfunction.

Participants demonstrated reduced gait cadence as MetS_index_ increased yet demonstrated large positive relationships between stance time and double limb support time MetS_index_. This hints that the mechanism underlying reduced gait velocity may be that participants took fewer steps—and not shorter steps—and thus were in contact with the ground for a longer duration during stance. Thus, future interventions may seek to focus on improving gait cadence as a means of increasing gait velocity in young adults with higher MetS_index_. Improved gait function by means of improved cadence may address exacerbating conditions that interfere with walking, such as reduced aerobic capacity and perceived discomfort and/or pain, improving participation and compliance with life-style modifications (18).

The results of this investigation should be considered in the context of notable limitations. The participants of this study were predominantly female. In our sample of 21 individuals, gait velocity was 1.16 m/s (females: 1.15 m/s, males: 1.17 m/s). Though the unequal distribution of sex between males and females could bias these results, there is a substantial body of literature which has established normative gait velocity data across different deciles of age (19, 20). Tolea et al., for example, demonstrated that preferred gait velocity between large samples of males (*N* = 401) and females (*N* = 595) under the age of 35 was comparable, at 1.2 m/s for both sexes (20). Given the established similarities between males and females in gait velocities of younger adults, we feel any bias due to unequal distribution of males and females is likely of minimal consequence on these results.

It is possible that the strength of the relationship between gait velocity and MetS_index_ may be moderated by additional anthropometric and functional components that impact both MetS_index_ and gait velocity but were not evaluated here. Thus, future endeavours may investigate these relationships on a larger sample, and consider additional anthropometric (e.g., weight distribution) and functional tests (e.g., balance, strength) that will provide additional data that can be used in further correlations and regressions to strengthen the relationship between function and MetS_index_. Likewise, determining functional limitations may also identify barriers impeding an individual's participation in physical activity interventions intended to minimize cardiometabolic disease risk.

## Data Availability

The raw data supporting the conclusions of this article will be made available by the authors, without undue reservation.
